# Spontaneous Coronary Artery Dissection Causing Cardiac Arrest in a Post-Partum Patient – A Case Report

**DOI:** 10.21980/J8F947

**Published:** 2021-10-15

**Authors:** Daniel Hoan Kim, Kevin O’Fee, Cindy C Bitter

**Affiliations:** * Saint Louis University School of Medicine, Division of Emergency Medicine, St. Louis, MO; ^ Washington University School of Medicine in St. Louis, Department of Internal Medicine, St. Louis, MO

## Abstract

Spontaneous coronary artery dissection is a rare condition that can precipitate acute coronary syndrome in pregnant and peripartum women.

A 32-year-old previously healthy female, 2-weeks post-partum, presented to the emergency department after sudden cardiac arrest. Return of spontaneous circulation was achieved after multiple rounds of resuscitation. Electrocardiogram revealed ST elevations in the anterolateral leads with reciprocal changes. She was taken to the catheterization lab by cardiology and was found to have a dissection of the distal left anterior descending coronary artery. The patient was subsequently treated medically with heparin and antiplatelet therapy, but ultimately died of anoxic encephalopathy.

The purpose of this report is to describe the risk factors, presentation, and management of spontaneous coronary artery dissection which can be easily overlooked given its rarity.

**Topics:**

Spontaneous coronary artery dissection, out-of-hospital cardiac arrest, peri-partum complications.

**Figure:**
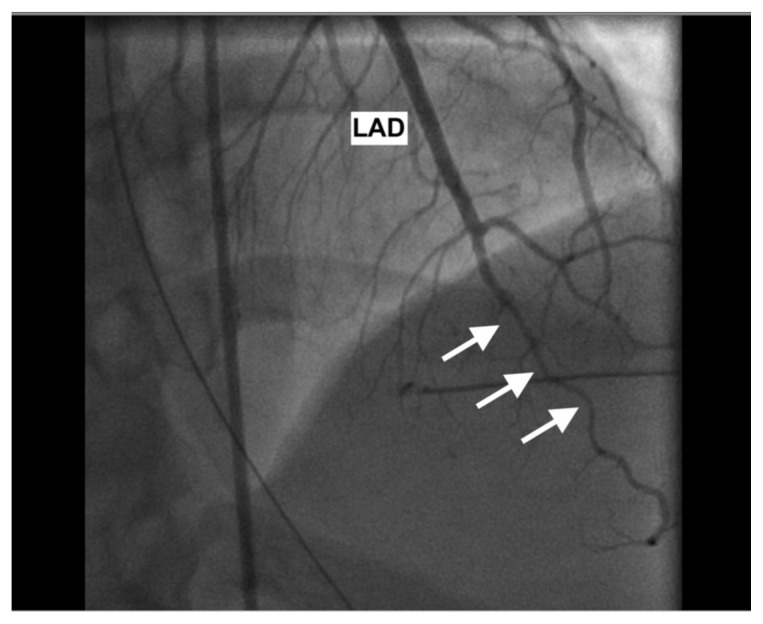


## Brief introduction

Spontaneous coronary artery dissection (SCAD) is a rare cause of acute coronary syndrome (ACS), but a frequent cause of myocardial infarction among pregnant and peripartum women.^1^,^2^ It is thought to be a result of coronary artery shear stress caused by hormonal and hemodynamic changes during pregnancy.^3^,^4^ Most frequently affected are post-partum women within the first month of giving birth, particularly in the first week.^5^ Although most of these patients do not carry typical cardiovascular risk factors, there is an increased risk in women with multiparity, infertility therapy, advanced age, and pre-eclampsia.^3^ Patients typically present with ACS including chest pain, dyspnea, and acute myocardial infarction.^6^ Cardiac arrest is the presenting symptom in 0–5% of these cases.^7^ Conservative medical management is preferred in stable dissections because most of these segments will heal spontaneously.^8^ However, more aggressive interventions such as percutaneous coronary intervention or coronary artery bypass surgery may be indicated depending on the need for prompt revascularization in the case of ongoing/recurrent ischemia or hemodynamic instability.^9^

## Presenting concerns and clinical findings

A 32-year-old female presented to the emergency department (ED) after becoming unresponsive in the backseat of her family’s car. Per family, the patient had suddenly fallen silent and appeared to be having seizure-like activity. Emergency medical services (EMS) was called and found the patient in cardiac arrest. The patient was intubated and cardiopulmonary resuscitation (CPR) was initiated, receiving three doses of epinephrine and four rounds of defibrillation on the way to the hospital. The patient arrived to the ED in pulseless ventricular fibrillation and CPR was continued. It was noted at this time that the patient was about two weeks post-partum. The patient’s mother recalled that the pregnancy had been uncomplicated. The patient had an obstetric history of six successful pregnancies and no prior history of cardiac disease, seizures, or eclampsia. Additionally, there was no family history of cardiac disease or sudden cardiac death. The patient was a known tobacco user but did not use alcohol or illicit drugs. She had not expressed any complaints in the days leading up to her arrest. Return of spontaneous circulation (ROSC) was achieved after a total of 35 minutes of resuscitation by EMS and the ED, which included multiple rounds of epinephrine, defibrillation, amiodarone, and magnesium.

## Significant findings

A post-ROSC electrocardiogram revealed ST elevations in leads I, aVL, and V3–V6, with reciprocal ST depressions in leads II, III, and aVF. Initial troponin I level was 0.238 ng/mL and a bedside cardiac ultrasound revealed decreased motion of the anterior wall. Cardiology was consulted and the patient was immediately taken to the catheterization lab where she was found to have long and diffuse luminal narrowing of her distal left anterior descending artery (LAD) resulting in 70% stenosis, consistent with the angiographic appearance of an intramural hematoma caused by dissection (white arrows). No intervention was performed.

## Patient course

The patient was admitted to the cardiac care unit and was treated with therapeutic heparin, aspirin, and clopidogrel. Given the history of seizure-like activity prior to arrest, a head CT was done which showed a global loss of gray white differentiation with signs of diffuse cerebral edema. A subsequent brain MRI performed 24 hours after arrest revealed diffuse hypoxic ischemic brain injury with impending herniation. The patient’s family was informed of these findings and the patient was placed on comfort care. She expired soon afterwards.

## Discussion

Spontaneous coronary artery dissection (SCAD) is challenging in terms of diagnosis and management. Patients with SCAD may lack typical risk factors for ACS, so a high index of suspicion is required. Prevalence of SCAD has been demonstrated as high as 4% in patients presenting with ACS.^2^ In women 50 years or younger, this number rises to 35% of all ACS cases.^1^

Classically, SCAD patients are described as middle-aged females with few, if any, cardiovascular risk factors. In a recent multicenter prospective observational study, women indeed comprised 89% of SCAD patients, with a mean age of 51.8+/− 10.2 years.^10^ SCAD also frequently occurs during pregnancy, with the majority of pregnancy-associated SCAD cases occurring post-partum, typically within the first week.^5^ However, pregnancy-associated SCAD has been found to constitute as low as 5% of SCAD cases overall.^10^ Other important risk factors include fibromuscular dysplasia, connective tissue disorders, and inflammatory conditions.^3^ Cases may also be precipitated by sympathomimetics and intense Valsalva maneuvers.^3^ Despite associations with certain predisposing conditions, SCAD has been observed from the 2^nd^ to 9^th^ decades of life and across a broad range of clinical presentations and risk factors.^10^

Diagnosis of SCAD is achieved by applying the same tools used for any suspected case of ACS. SCAD involves the acute development of a hematoma within the tunica media (false lumen), which leads to compression of the true coronary vessel lumen.^11^ Sex hormones, which are known to alter arterial composition during pregnancy, likely also play a role in the development of SCAD by altering hemodynamics and increasing shear forces against the coronary arterial wall.^3^,^4^ Though distinct from the mechanism of unstable atherothrombotic plaque rupture that defines most cases of myocardial infarction, the end result of SCAD is the same - occlusion of an epicardial coronary artery leading to ischemia and subsequent myocardial infarction.^11^ Thus the mainstay of workup remains the pursuit of anginal symptoms or equivalents in the patient history, detection of dynamic EKG changes concerning for ischemia and infarction such as new ST-segment changes or Q waves, and rise and/or fall of serial troponin biomarkers for establishing a diagnosis of ACS due to SCAD.^12^

A positive evaluation for ACS should prompt activation of the cardiac catherization lab. Generally, the presence of coronary tortuosity in the absence of intracoronary thrombus are angiographic hints to the presence of SCAD.^3^

The goals in the management of SCAD align with that of atherothrombotic etiologies of myocardial infarction – restoring blood flow to coronary arteries and reperfusion of the myocardium. However, key differences in the means of achieving this goal exist. First, thrombolytics are generally recommended against because they can increase hematoma and dissection size potentially leading to deterioration of clinical status.^13^ Similarly, anti-coagulation agents and glycoprotein IIb/IIIa inhibitors must be used cautiously because they also carry the theoretical risk of dissection propagation.^9^ To date, no evidence has demonstrated a significant benefit in the routine employment of any of these agents in the management of SCAD.^3^

As in other cases of ACS, percutaneous coronary intervention (PCI) is a therapeutic option in SCAD management. However, SCAD constitutes a dynamic coronary injury, and hematoma propagation occurs in up to 1/3 of SCAD cases.^14^ This leads to unpredictable PCI results often rife with complications such as iatrogenic dissection.^15^ In conservatively managed patients (PCI deferred), observational studies have demonstrated that spontaneous “healing” of SCAD lesions occur in a majority of patients, most often within 30 days.^8^ Recent guidelines have echoed this evidence by recommending conservative treatment whenever possible.^9^ However, in instances of ongoing ischemia, left main artery dissection, or hemodynamic instability, PCI or even coronary artery bypass grafting (CABG), should be strongly considered.^9^

Although coronary angiography necessitates radiation exposure, pregnant women presenting with high-risk features of ACS or SCAD should still be treated according to the standard of care for acute myocardial infarction.^16^ The degree of radiation is low especially with shielding, and risk of potential cardiovascular collapse and death for both mother and fetus is high.^17^ Other important considerations for emergency department management include utilizing the left lateral recumbent position to optimize venous return, considering the use of continuous fetal monitoring especially in the viable fetus, and considering the use of antenatal corticosteroids if pre-term delivery is anticipated within days.^6^ It is preferable to wait 14 days between acute SCAD and delivery. However, if this is impossible, optimization of maternal cardiac status followed by planned delivery under controlled conditions by a multidisciplinary team may become necessary.^6^

Unfortunately, SCAD patients experience a high frequency of recurrent SCAD, which can occur as a new dissection, or extension of an old dissection. Rates of recurrence range from 10% to 30% and most commonly occur within the first 7 days of presentation.^10^,^18^ In a large, single-center, prospective, non-randomized observational study, beta blockers were found to reduce the risk of major adverse cardiovascular events (MACE) following SCAD, whereas anti-platelet and statins did not significantly affect outcomes. Poorly controlled hypertension significantly increased the risk of MACE.^19^ Because pregnancy carries a risk of recurrence, highly effective contraception is recommended for women of child-bearing age, such as IUDs or permanent sterilization in the case of women who have completed their family. For hormonal contraception methods, estrogen-containing options are preferably avoided because high estrogen states are associated with SCAD.^20^

SCAD truly presents a diagnostic and therapeutic challenge. Among SCAD patients, it is important to remember the wide spectrum of possible risk and clinical phenotypes at presentation, the need for rapid ACS evaluation and timely activation of the cardiac catheterization lab, to avoid thrombolytics, to watch closely for high risk features such as ongoing ischemia or hemodynamically instability, to pay special attention in the care of pregnant women, and to be mindful of the high risk of recurrence.

## Supplementary Information


