# Supplementary figures and images for: Spontaneous Coronary Artery Dissection Causing Cardiac Arrest in a Post-Partum Patient – A Case Report
Source: J Educ Teach Emerg Med. 2021 Oct 15;6(4):V1–5. doi: 10.21980/J8F947 (PMC10332733; doi:10.21980/J8F947)

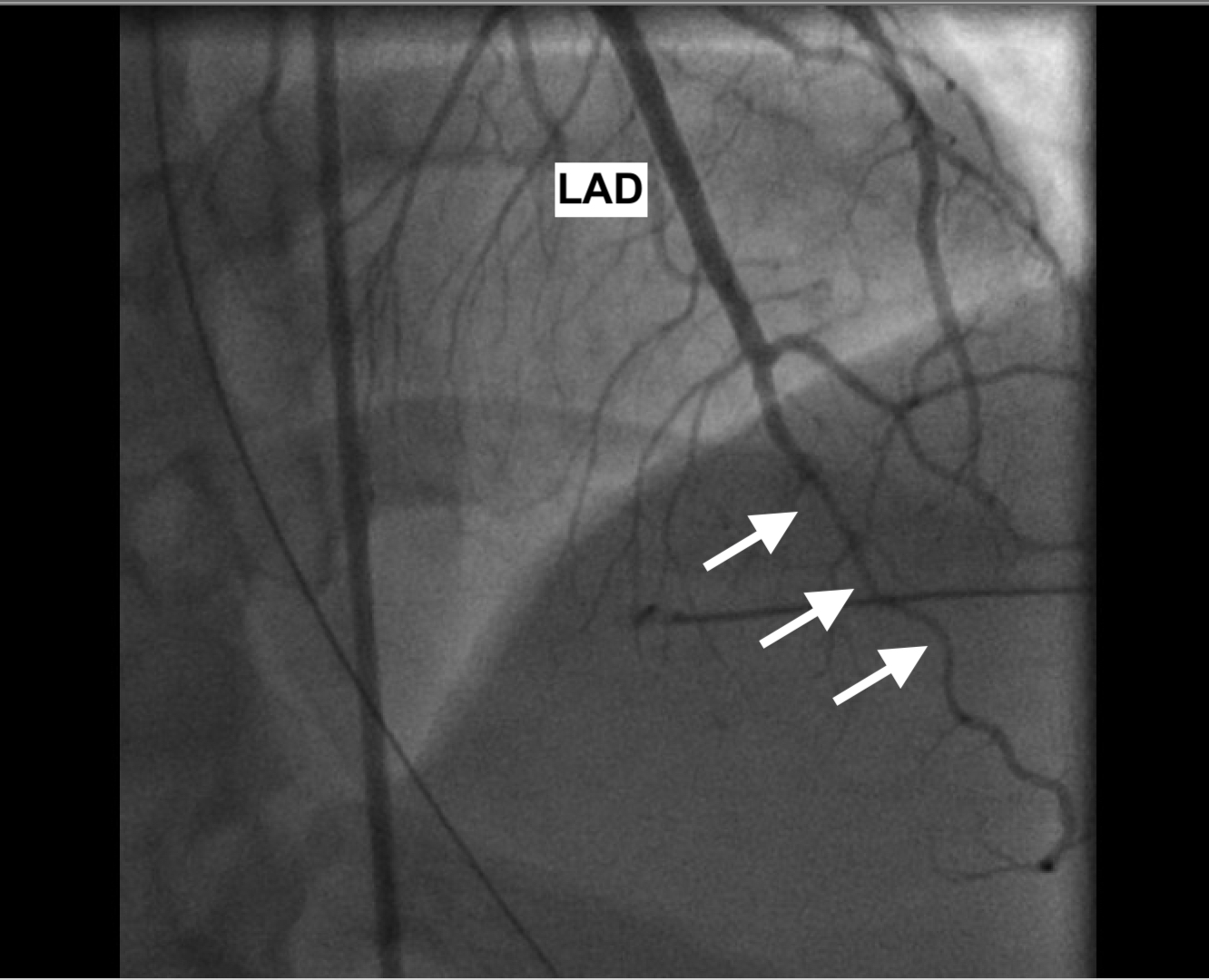

Supplement: Supplementary file 1 [file JETem-6-4-V1-supp1.jpg]
